# INTEGRATING HOUSING, HEALTH, AND SUPPORTIVE CARE IN AFFORDABLE SENIOR HOUSING: EVALUATION OF THE R3 PROGRAM

**DOI:** 10.1093/geroni/igac059.819

**Published:** 2022-12-20

**Authors:** Edward Miller, Marc Cohen

## Abstract

This symposium reports evaluation findings from the Right Care, Right Place, Right Time (R3) program. The initiative is designed to integrate housing, health, and supportive care to residents of affordable senior housing using a wellness team (nurse and social worker). The embedded team works directly with residents to address health-related, educational, and informational needs and access to services - focusing on proactive outreach and prevention, coordination with providers, constant contact with residents, and targeting high-risk residents. The initiative aims to create a replicable, scalable, and sustainable model of housing with supportive services that enables independent living while reducing health care costs. Two wellness teams served approximately 400 participants at seven Boston-area buildings. The R3 program was implemented in two phases. The 18-month pre-intervention period was January 2016-March 2017, while the 18-month Phase 1 intervention period was July 2017-December 2018. The 21-month Phase 2 implementation period (known R32), which introduced a targeting strategy to identify high risk residents, was January 2019-September 2020. Evaluation activities included quantitative and qualitative components. Program participants and non-participants at intervention and comparison sites were surveyed on program-related experiences. Program, housing, and community partners were interviewed. Key performance indicators were tracked. Medicare claims data were analyzed using comparison groups. Focus groups were completed with payers, housing providers, and community stakeholders. The purpose of this symposium is to identify the experiences of program participants and key stakeholders with the R3 program, and to assess program impact. Edward Miller and Marc Cohen will serve as chair and co-chair, respectively.

